# Interdisciplinary Management of Severe Excessive Gingival Display: A Case Report

**DOI:** 10.7759/cureus.112168

**Published:** 2026-07-06

**Authors:** Meriem Bellamine, Amadou Oury Diallo, François Tonamou, Chaymae Chafik

**Affiliations:** 1 Orthodontics and Dentofacial Orthopaedics, Faculty of Dental Medicine, Mohammed VI University of Health Sciences, Casablanca, MAR

**Keywords:** excessive gingival display, genioplasty, gingivectomy, gummy smile, interdisciplinary treatment, maxillofacial surgery orthognathic surgery, skeletal class ii malocclusion, vertical maxillary excess

## Abstract

Excessive gingival display (EGD) associated with hyperdivergent skeletal Class II malocclusion represents a complex clinical condition requiring comprehensive interdisciplinary management. Successful treatment depends on accurate identification of the underlying skeletal, dentoalveolar, periodontal, and soft-tissue etiologic factors, combined with close coordination between orthodontic, orthognathic, and periodontal procedures. This case report describes the interdisciplinary management of severe EGD in an adult patient using presurgical orthodontics, bimaxillary orthognathic surgery, secondary genioplasty, and adjunctive gingivectomy, highlighting the benefits of a multidisciplinary approach in achieving favorable functional and esthetic outcomes with long-term stability.

A 26-year-old female patient presented with hyperdivergent skeletal Class II malocclusion, associated with severe EGD resulting from vertical maxillary excess, a short upper lip, and localized gingival hypertrophy, and was treated using a sequential interdisciplinary approach. Treatment began with presurgical orthodontics, followed by bimaxillary orthognathic surgery consisting of Le Fort I maxillary impaction combined with bilateral sagittal split mandibular advancement osteotomy. Secondary advancement and vertical reduction genioplasty was subsequently performed to optimize lower facial balance and compensate for the anatomical limitations of mandibular advancement. Because localized gingival hypertrophy persisted after correction of the skeletal component of the EGD, an adjunctive 2-mm periodontal gingivectomy was performed, increasing the clinical crown length of the maxillary central incisors from 7 mm to 9 mm and further improving smile esthetics. Treatment resulted in a reduction of the gingival display from 8 mm preoperatively to ≤3 mm post-treatment, improvement of ANB from 9° (presurgical) to 5° (post-treatment) and GoGn/SN from 48° to 43°, establishment of a stable bilateral Class I occlusion, and satisfactory smile esthetics. The three-year and eight-month follow-up confirmed favorable esthetic and functional stability, with no significant relapse observed.

An interdisciplinary approach combining orthodontic treatment, orthognathic surgery, and adjunctive periodontal management represents an effective treatment option for complex cases of EGD, allowing predictable esthetic and functional outcomes with long-term stability.

## Introduction

Excessive gingival display (EGD), commonly referred to as a gummy smile, is a significant esthetic concern that may negatively affect facial harmony and psychosocial well-being. Gingival exposure greater than 3 mm during smiling is generally considered excessive and frequently motivates patients to seek treatment [1-3]. The etiology of EGD is multifactorial and may involve skeletal factors, such as vertical maxillary excess, dentoalveolar factors including altered passive eruption, and soft-tissue factors such as a short or hyperactive upper lip [2,4].

Because of this complexity, management often requires an interdisciplinary approach involving orthodontics, maxillofacial surgery, and periodontics [5,6]. Orthognathic surgery, particularly Le Fort I maxillary impaction, remains the treatment of choice for severe skeletal gummy smiles associated with vertical maxillary excess. However, when localized gingival hypertrophy persists after correction of the skeletal component, adjunctive periodontal procedures, including gingivectomy, may be required to expose the anatomical crowns, improve dentogingival harmony, and optimize smile esthetics [7,8].

Despite the availability of established treatment modalities, managing EGD of multifactorial origin remains clinically challenging because each etiologic component requires a specific therapeutic approach and careful interdisciplinary coordination. Furthermore, reports describing comprehensive sequential interdisciplinary management with long-term follow-up remain limited.

This case report describes the sequential interdisciplinary management of an adult patient presenting with hyperdivergent skeletal Class II malocclusion associated with severe EGD resulting from vertical maxillary excess, a short upper lip, and localized gingival hypertrophy. It highlights the successful integration of presurgical orthodontics, bimaxillary orthognathic surgery, secondary genioplasty, and adjunctive periodontal treatment, demonstrating how a multidisciplinary approach can achieve stable long-term functional and esthetic outcomes.

## Case presentation

A 26-year-old female patient (Y.K.) in apparent good general health, with no significant medical or surgical history, no known drug allergies, and a non-smoker, presented with a major esthetic concern related to EGD during smiling associated with an anteroposterior skeletal discrepancy.

**Figure 1:**
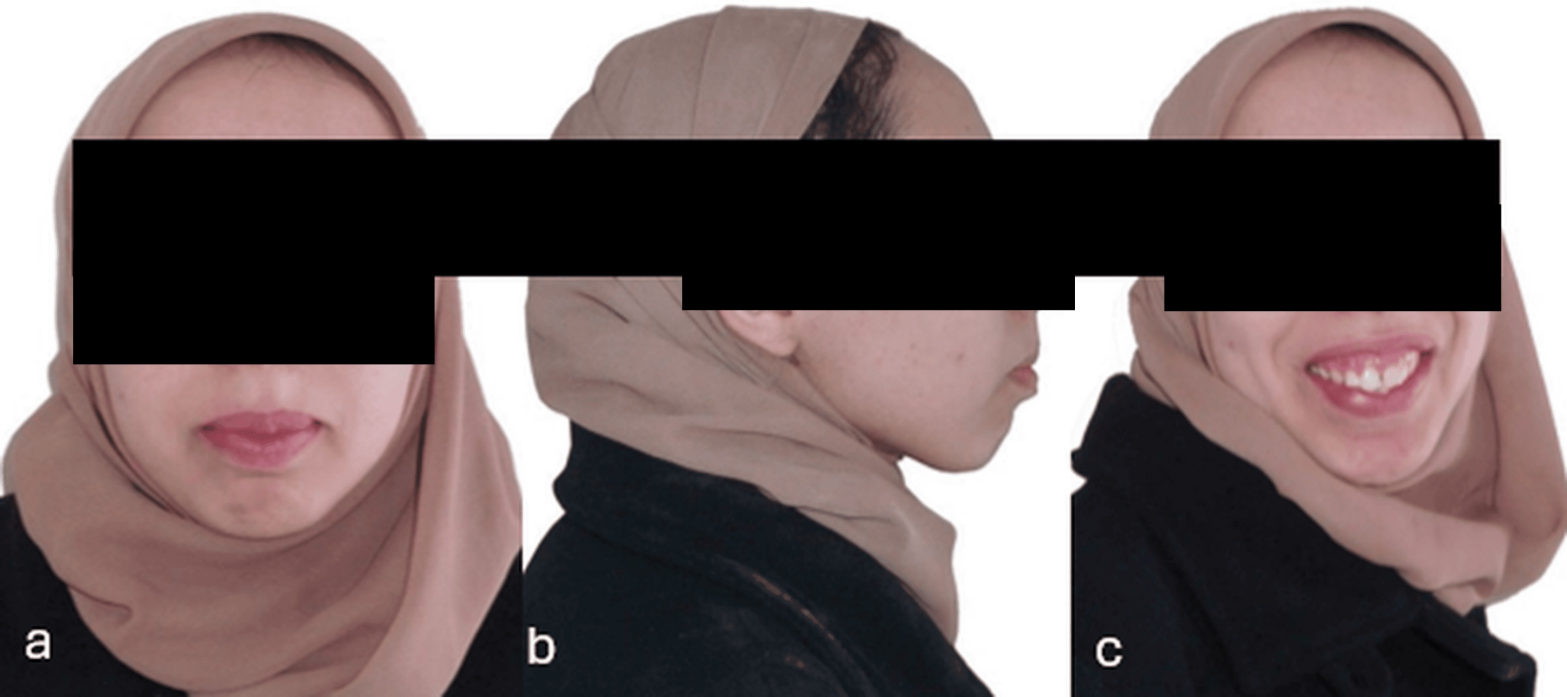
Pre-treatment extraoral photographs. (a) Frontal view at rest. (b) Right profile view. (c) Smiling frontal view demonstrating severe excessive gingival display.

 Clinical examination revealed increased lower facial height, lip incompetence requiring active lip closure, and an increased nasolabial angle. Smile evaluation demonstrated severe and symmetrical EGD of approximately 7-8 mm at the level of the maxillary central incisors, associated with a short upper lip. Profile analysis showed a convex facial profile with relative retrogenia, consistent with a skeletal Class II malocclusion (Figures 1a-1c).

**Figure 2:**
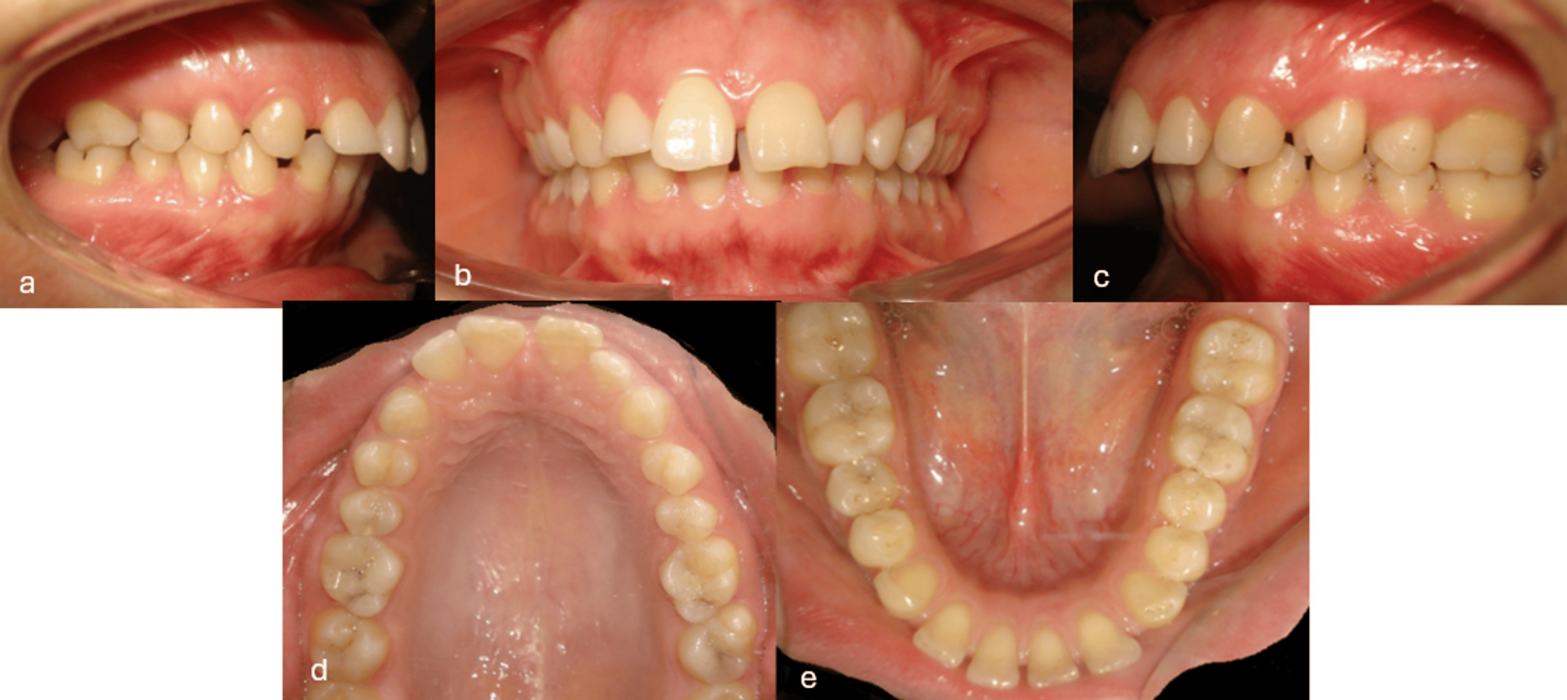
Pre-treatment intraoral records. (a) Right lateral intraoral view. (b) Frontal intraoral view. (c) Left lateral intraoral view. (d) Maxillary occlusal view. (e) Mandibular occlusal view.

**Figure 3:**
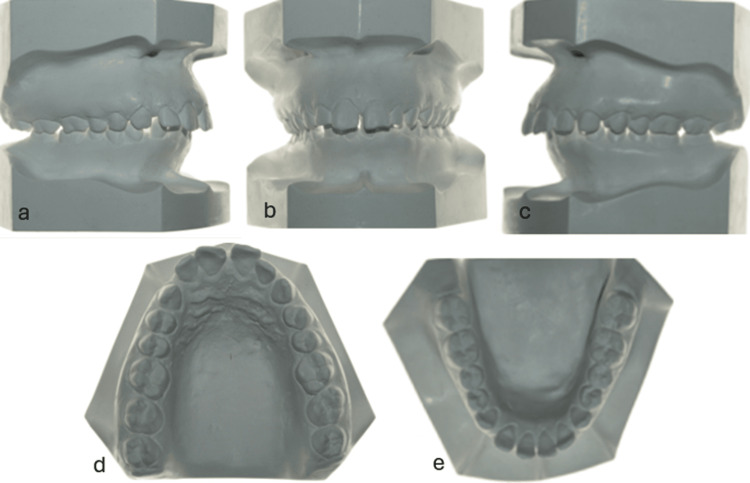
Pre-treatment study models showing the initial occlusal relationships. (a) Right lateral intraoral view. (b) Frontal intraoral view. (c) Left lateral intraoral view. (d) Maxillary occlusal view. (e) Mandibular occlusal view.

Intraoral examination revealed a complete permanent dentition with bilateral full-cusp Class II molar and canine relationships. The overjet measured 8 mm and the overbite 5 mm. Generalized spacing was observed in both the maxillary and mandibular arches, associated with marked dentoalveolar protrusion (Figures 2a-2e and Figures 3a-3e).

**Figure 4:**
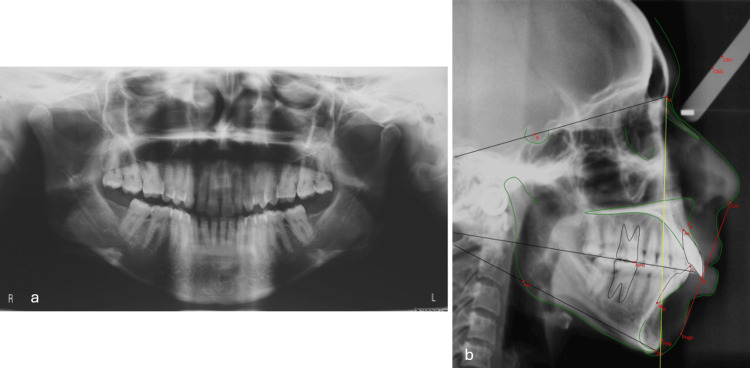
Pre-treatment radiographic records. (a) Panoramic radiograph. (b) Lateral cephalometric radiograph.

Lateral cephalometric analysis demonstrated a hyperdivergent skeletal Class II pattern characterized by mandibular retrusion, an increased mandibular plane angle, and pronounced mandibular incisor protrusion (Figures 4a-4b; Table 1).

**Table 1:** Cephalometric measurements at initial assessment, presurgical stage, and post-treatment.

Cephalometric variable	Pre-treatment	Presurgical orthodontic phase	Post-treatment
SNA (°)	80	82	82
SNB (°)	74	73	77
SND (°)	70	68	73
ANB (°)	6	9	5
GoGn/SN (°)	45	48	43
U1/NA (°)	20	16	19
U1-NA (mm)	4	2	3
L1/NB (°)	40	35	37
L1-NB (mm)	8	7	7
I/i (°)	113	133	121
Pog/NB (mm)	5	5	3

Based on the clinical and radiographic findings, the patient presented with a hyperdivergent skeletal Class II malocclusion (ANB = 6°) associated with mandibular retrusion and an increased mandibular plane angle (GoGn/SN = 45°). Dentally, bilateral full-cusp Class II canine and molar relationships, increased overjet and overbite, generalized spacing, and marked mandibular dentoalveolar protrusion were observed. In addition, the patient exhibited severe EGD with skeletal, soft-tissue, and periodontal components, including vertical maxillary excess, a short upper lip, and mild gingival hypertrophy (Figures 1-2; Table 1).

The treatment plan was established through close collaboration between the orthodontist, maxillofacial surgeon, and periodontist, in accordance with the principles of interdisciplinary management of complex dentofacial deformities. This integrated approach aimed to simultaneously achieve comprehensive and harmonious esthetic, functional, and structural outcomes.

**Figure 5:**
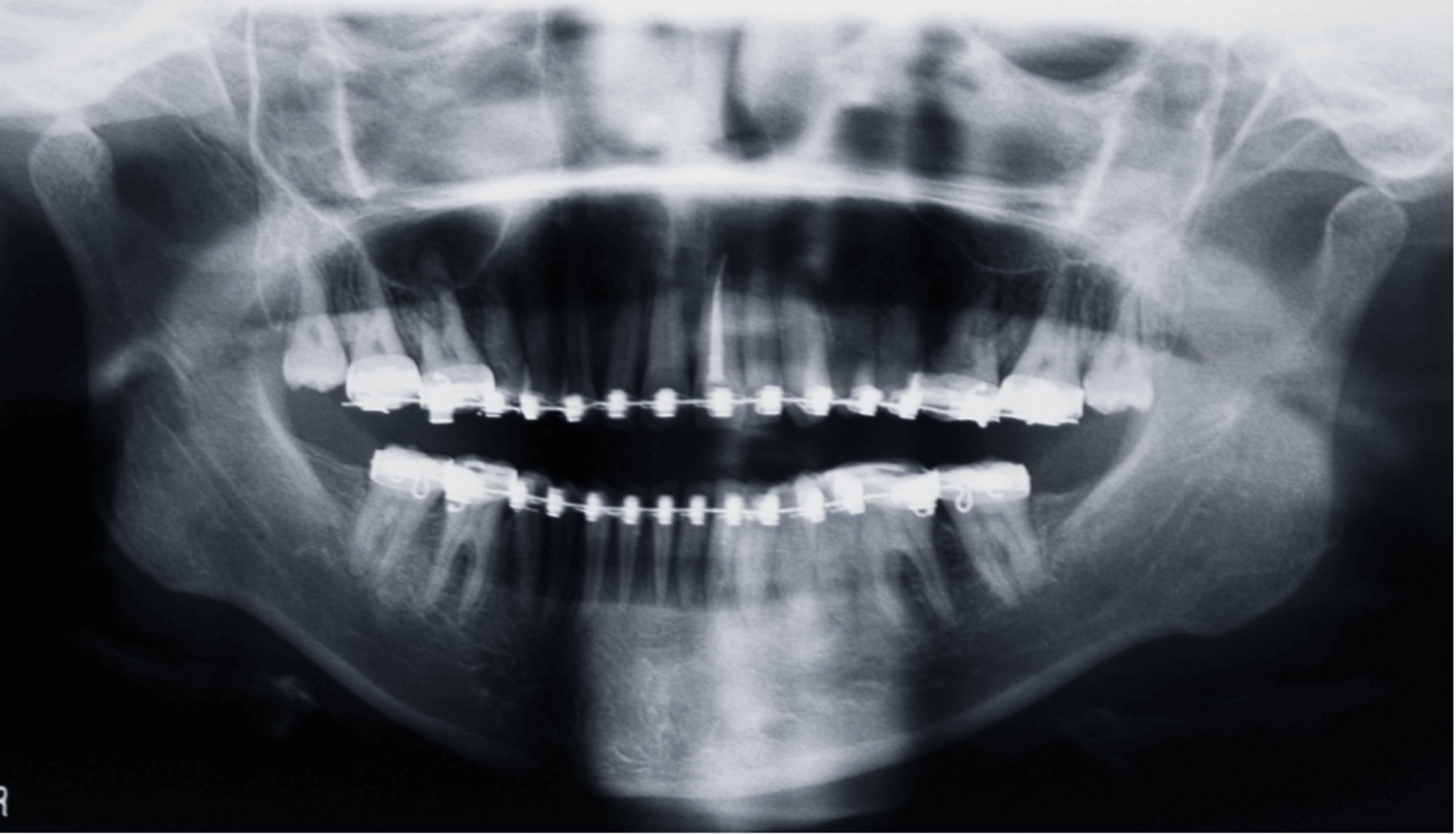
Presurgical panoramic radiograph.

**Figure 6:**
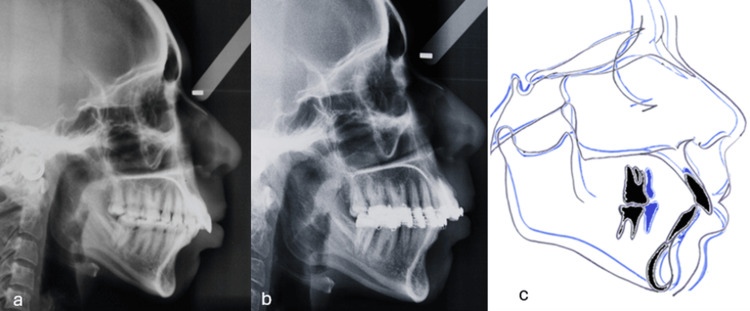
Lateral cephalometric radiographs. (a) Pre-treatment. (b) Presurgical after orthodontic decompensation. (c) Superimposition of pre-treatment (black) and presurgical (blue) tracings.

Presurgical orthodontic treatment was carried out over approximately 12 months using standard fixed multibracket appliances in both arches. The objectives of this phase were to align and level the dental arches, close residual diastemas, eliminate dental compensations, and coordinate the arches in preparation for orthognathic surgery. Controlled orthodontic mechanics were used throughout treatment to achieve dentoalveolar decompensation while maintaining the planned vertical dimension and avoiding undesirable tooth extrusion. Adequate root parallelism, coordinated arches, and stable intercuspation were achieved before surgery. Passive rectangular stainless-steel surgical archwires were placed four to six weeks before surgery to stabilize tooth positions and facilitate intraoperative guidance. Presurgical radiographic assessment and study model analysis confirmed satisfactory preparation for surgical intervention (Figure 5 and Figures 6a-6c).

**Figure 7:**
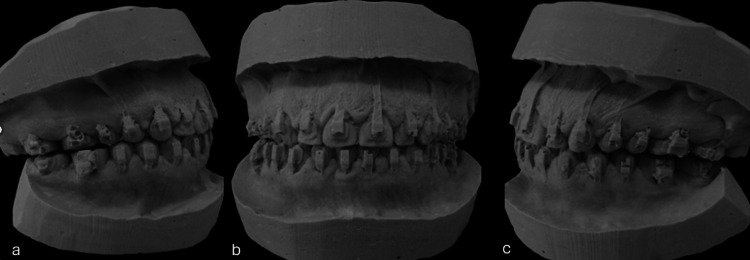
(a-c) Presurgical study models in occlusion used for surgical planning.

Presurgical planning was performed using study models in occlusion, allowing visualization of the required skeletal movements and fabrication of surgical splints (Figures 7a-7c).

**Figure 8:**
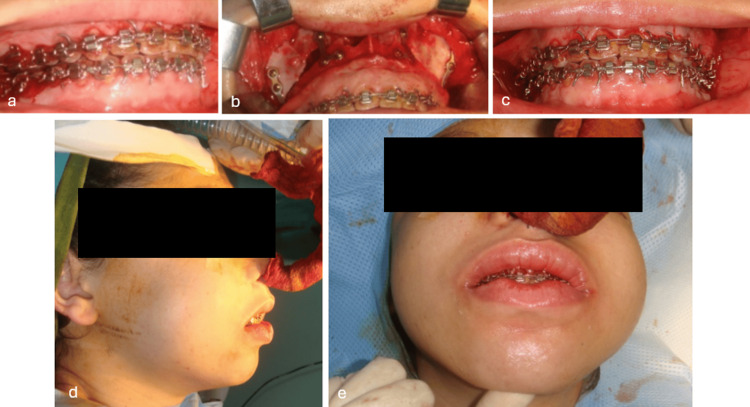
Intraoperative views of bimaxillary orthognathic surgery. (a) Right buccal intraoral view after maxillary repositioning. (b) Le Fort I osteotomy with maxillary impaction and rigid internal fixation. (c) Frontal intraoral view following maxillary fixation. (d) Lateral intraoperative facial assessment after maxillary impaction and mandibular advancement. (e) Frontal intraoperative facial assessment following bimaxillary repositioning.

Orthognathic surgery was performed under general anesthesia following completion of the presurgical orthodontic phase. The surgical procedure consisted of a Le Fort I osteotomy with 5 mm of superior maxillary impaction, without concomitant maxillary setback, to correct vertical maxillary excess, reduce EGD, and improve vertical facial proportions. Mandibular retrognathia was corrected through bilateral sagittal split osteotomy (BSSO) advancement to achieve an edge-to-edge incisal relationship, resulting in mandibular autorotation and thereby contributing to the establishment of a skeletal Class I relationship. Skeletal stabilization was achieved using rigid internal fixation with titanium miniplates and screws (Figures 8a-8c).

**Figure 9:**
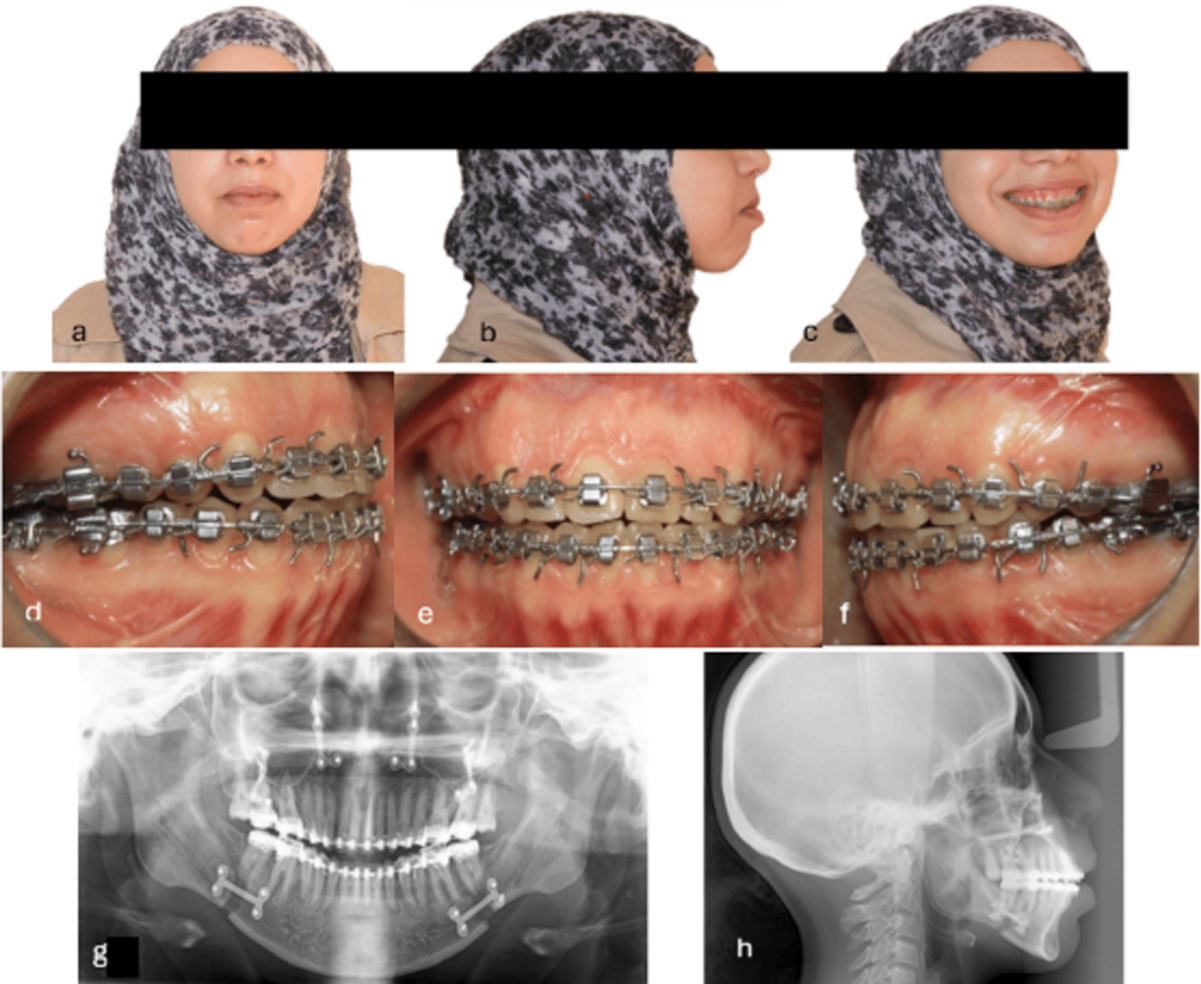
Post-surgical records at two-month follow-up. (a) Frontal extraoral photograph. (b) Right profile extraoral photograph. (c) Smiling extraoral photograph. (d) Right buccal intraoral view. (e) Frontal intraoral view. (f) Left buccal intraoral view. (g) Panoramic radiograph. (h) Lateral cephalometric radiograph.

**Figure 10:**
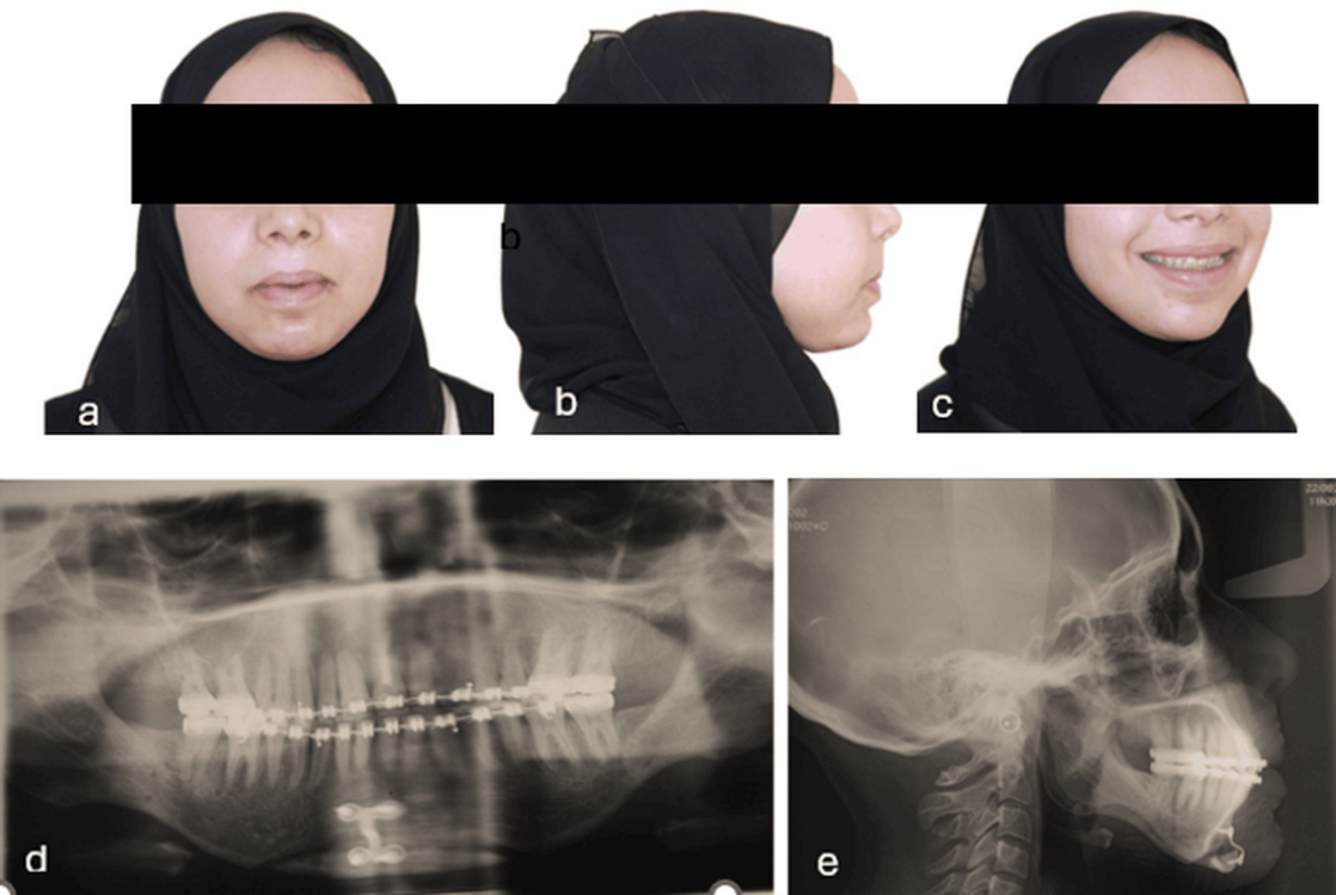
Postoperative assessment following advancement genioplasty and osteosynthesis plate removal. (a) Frontal extraoral photograph. (b) Right profile extraoral photograph. (c) Smiling extraoral photograph. (d) Panoramic radiograph obtained after plate removal. (e) Lateral cephalometric radiograph showing the skeletal and soft tissue changes achieved following advancement genioplasty.

A secondary genioplasty was performed concomitantly with osteosynthesis plate removal, six months after the initial orthognathic surgery, once postoperative skeletal stability had been confirmed. Despite the favorable skeletal correction achieved after the bimaxillary procedure, residual chin retrusion and inadequate lower facial projection persisted, compromising facial harmony (Figures 9a-9h). Therefore, an advancement and downward genioplasty was undertaken to enhance chin projection, improve lower facial proportions, and optimize the cervicomental contour and overall facial esthetics (Figures 10a-10e).

**Figure 11:**
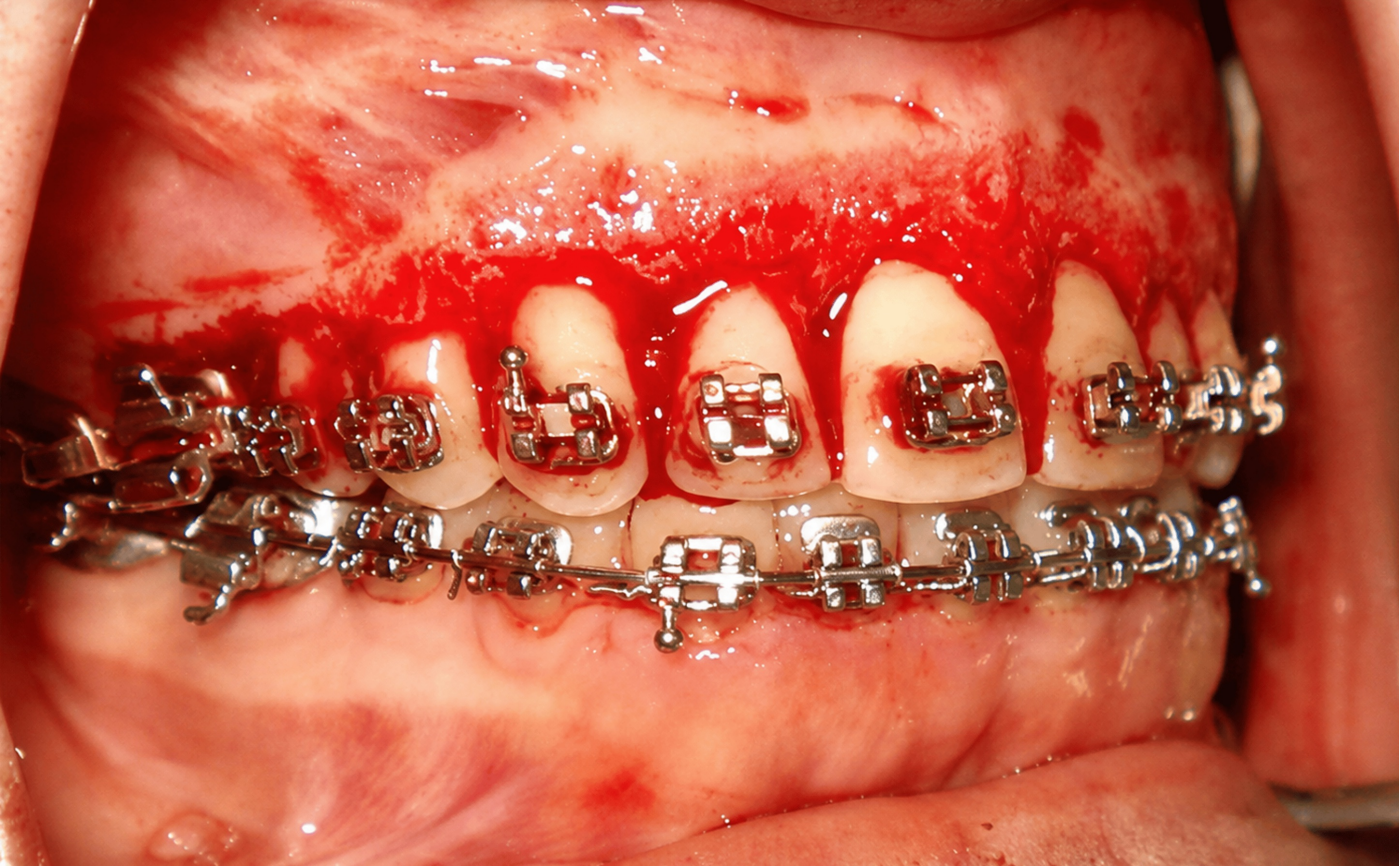
Clinical intraoperative photograph showing gingivectomy performed before orthodontic appliance removal to improve gingival contour and optimize smile esthetics.

**Figure 12:**
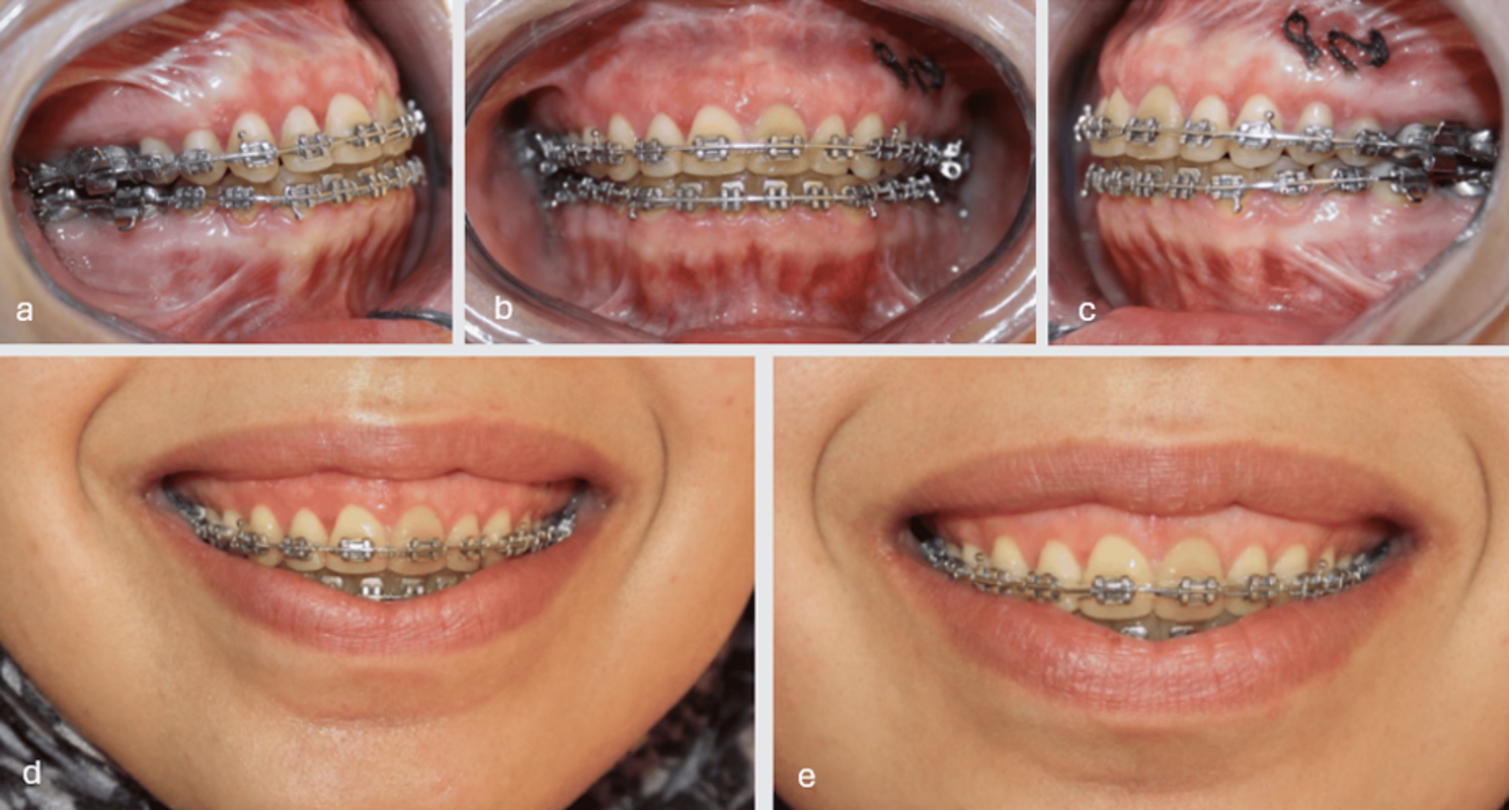
Finishing the orthodontic phase before appliance removal. (a) Right buccal intraoral view. (b) Frontal intraoral view. (c) Left buccal intraoral view. (d) Frontal smile photograph. (e) Frontal smile photograph demonstrating the final smile aesthetics prior to appliance removal.

As part of the finishing phase, a comprehensive periodontal evaluation confirmed healthy periodontal tissues with localized gingival hypertrophy in the maxillary anterior region. Although orthognathic surgery successfully corrected the skeletal component of the EGD, localized gingival hypertrophy persisted, partially covering the anatomical crowns and contributing to the residual EGD. Therefore, a 2-mm gingivectomy was performed before debonding, increasing the clinical crown length of the maxillary central incisors from 7 mm to 9 mm. This adjunctive periodontal procedure improved dentogingival harmony, enhanced gingival symmetry, and optimized the final smile esthetics (Figure 11). Subsequently, the finishing orthodontic phase allowed optimization of intercuspation and final occlusal settling (Figures 12a-12e). Fixed multibracket appliances were removed after a total treatment duration of 24 months. Teeth 18 and 28 were then extracted, and a strict retention protocol was implemented, consisting of bonded lingual fixed retainers combined with removable clear retainers to ensure long-term occlusal stability.

Results

**Figure 13:**
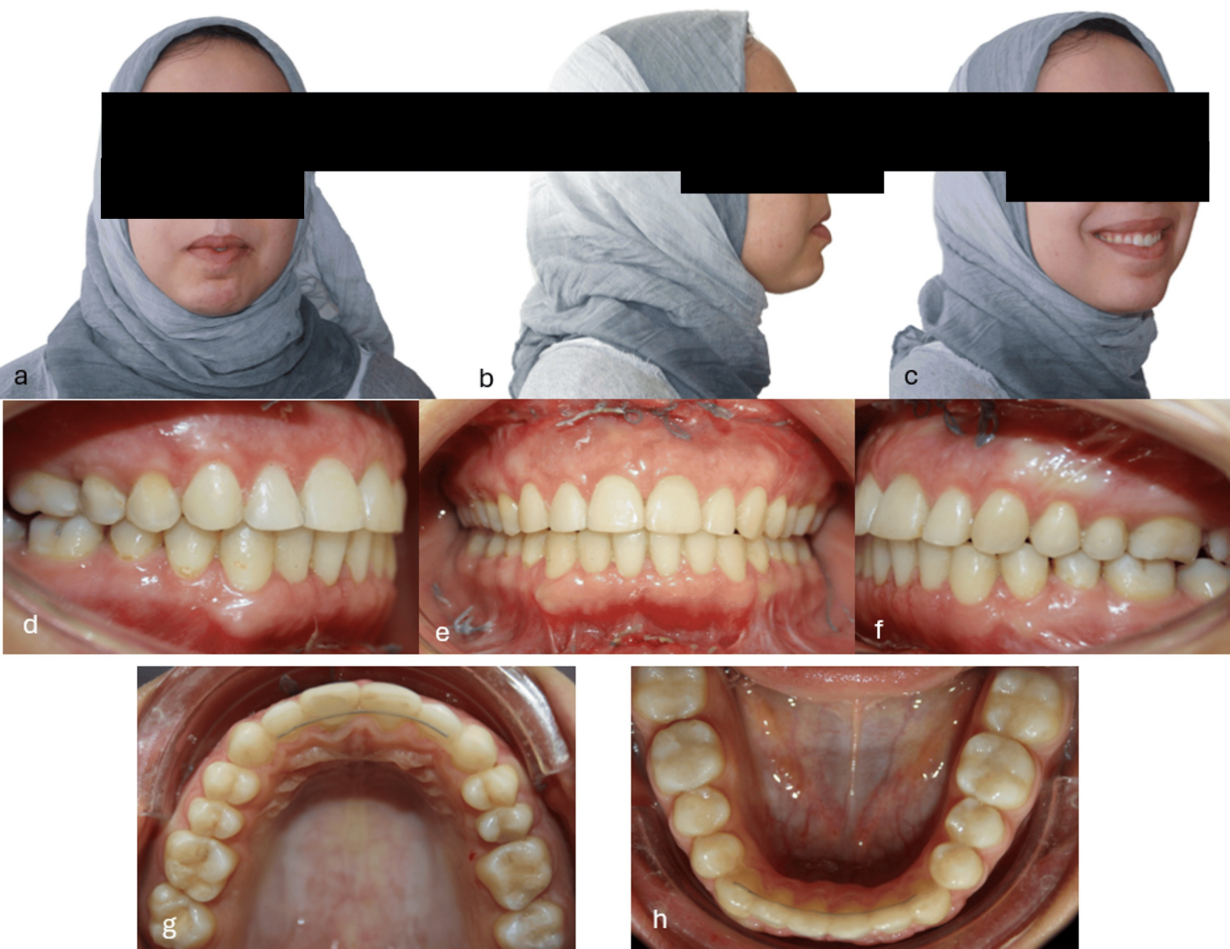
Post-treatment records. (a) Frontal extraoral photograph. (b) Right profile extraoral photograph. (c) Smiling extraoral photograph. (d) Right buccal intraoral view. (e) Frontal intraoral view. (f) Left buccal intraoral view. (g) Maxillary occlusal view. (h) Mandibular occlusal view showing the bonded lingual retainer.

**Figure 14:**
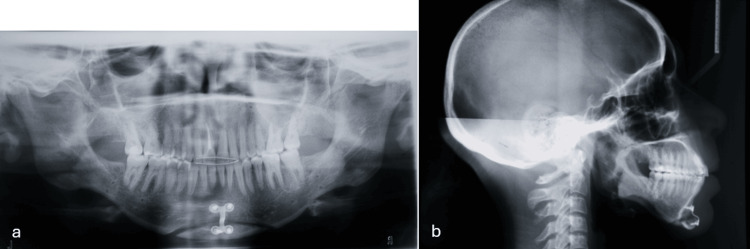
Post-treatment records. (a) Panoramic radiograph. (b) Lateral cephalometric radiograph.

**Figure 15:**
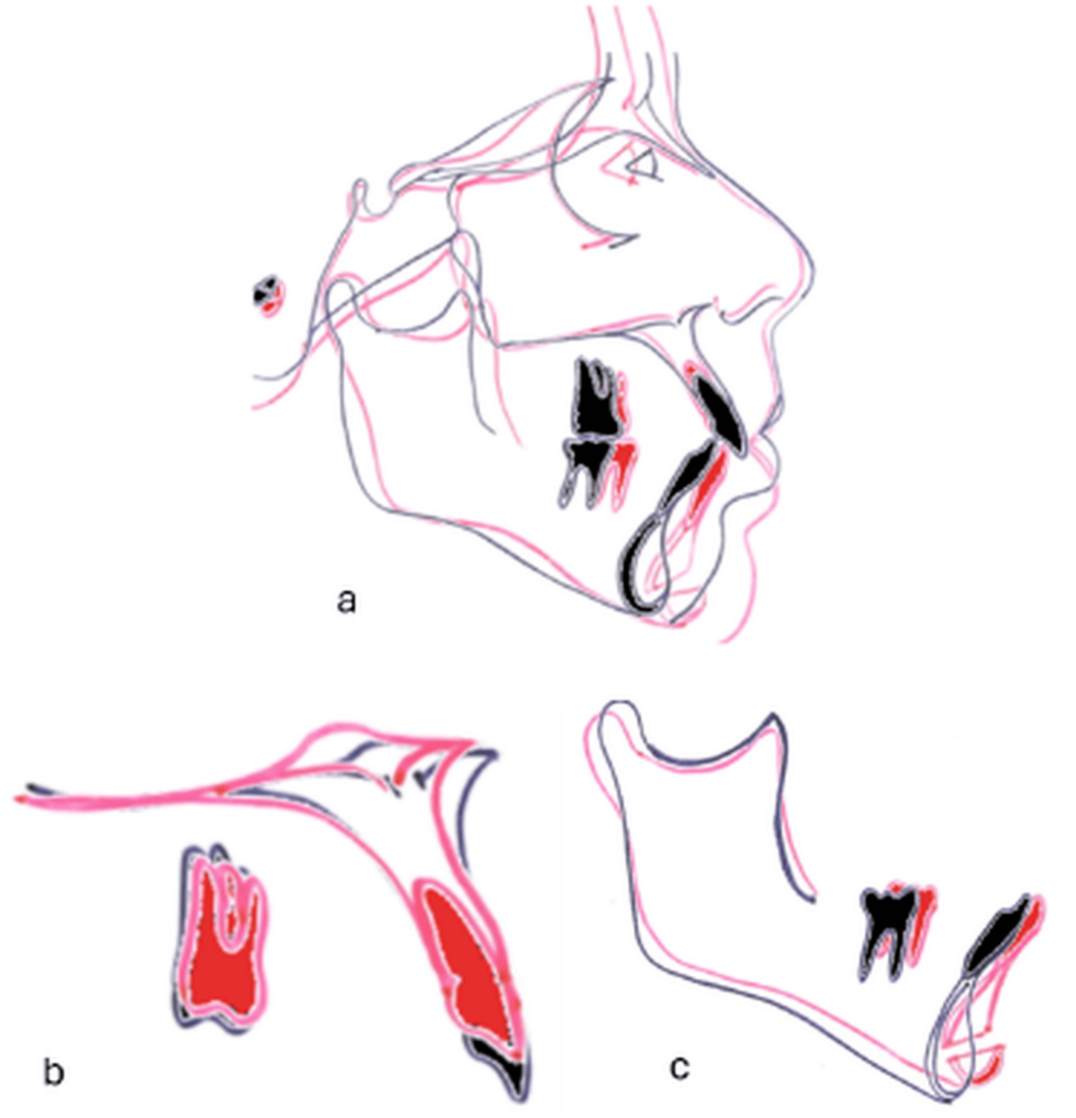
Cephalometric superimpositions illustrating the skeletal changes following treatment. (a) Overall superimposition. (b) Maxillary superimposition demonstrating superior repositioning of the maxilla after Le Fort I impaction. (c) Mandibular superimposition showing mandibular advancement with counterclockwise autorotation following bilateral sagittal split osteotomy (BSSO) and advancement genioplasty.

Treatment resulted in favorable improvements, with ANB decreasing from 9° at the presurgical stage to 5° post-treatment and GoGn/SN decreasing from 48° to 43°, resulting in a more harmonious facial profile (Table 1). From an occlusal perspective, a functional bilateral Class I occlusion was achieved, with optimal overjet and overbite values of 2 mm. In addition, EGD, initially measured at 8 mm, was reduced to ≤3 mm during forced smiling, thereby fulfilling the aesthetic objectives of treatment. This outcome was achieved through a sequential interdisciplinary approach, in which maxillary impaction corrected the skeletal component of EGD, while the adjunctive 2-mm gingivectomy increased the clinical crown length of the maxillary central incisors from 7 mm to 9 mm, improving dentogingival harmony, gingival symmetry, and smile esthetics (Figures 13a-13h, Figures 14a-14b, and Figures 15a-15c).

Following appliance removal, fixed bonded retainers were placed in both the maxillary and mandibular arches, and removable clear retainers were prescribed for nightly wear. Teeth 18 and 28 were extracted at the completion of treatment. The patient was enrolled in a regular follow-up program to monitor occlusal stability, periodontal health, and smile esthetics.

The subjective perception of treatment success was further supported by the Orthognathic Quality of Life Questionnaire (OQLQ) [9], administered before treatment and after completion of treatment. The OQLQ score decreased from 86/88 before treatment to 4/88 after treatment, reflecting a marked improvement in oral health-related quality of life. The greatest improvements were observed in the domains of facial esthetics and social functioning, highlighting the positive psychosocial impact of the interdisciplinary treatment (Table 2).

**Table 2:** Orthognathic Quality of Life Questionnaire (OQLQ) scores before and after treatment.

Domain	Pre-treatment	Post-treatment
Facial aesthetics	20/20	2/20
Oral function	18/20	0/20
Awareness of facial deformity	16/16	2/16
Social aspects	32/32	0/32
Total OQLQ score	86/88	4/88

**Figure 16:**
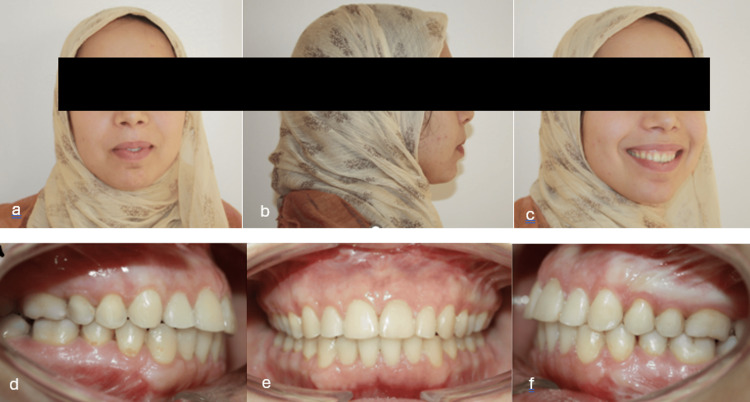
Long-term follow-up records at three years and eight months. (a-c) Extraoral photographs. (d-f) Intraoral photographs demonstrating stable esthetic and occlusal outcomes.

At the three-year and eight-month follow-up, the treatment results appeared to remain stable both skeletally and occlusally. A bilateral Class I occlusion was maintained, with satisfactory intercuspation and preservation of facial balance. Gingival display remained within acceptable aesthetic limits, without evidence of clinically significant relapse of the gummy smile or recurrence of the underlying skeletal discrepancy. Overall, facial harmony and smile aesthetics were preserved over time (Figures 16a-16f).

**Figure 17:**
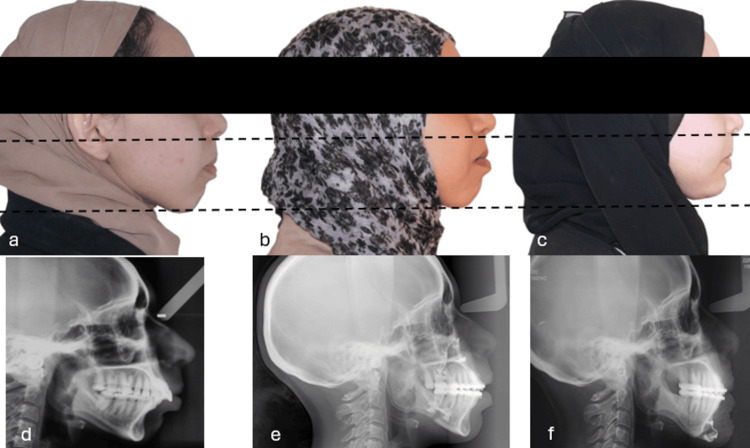
Treatment progression. (a) Pre-treatment profile photograph. (b) Profile photograph after bimaxillary orthognathic surgery. (c) Final profile photograph after secondary genioplasty and completion of treatment. (d) Pre-treatment lateral cephalometric radiograph. (e) Lateral cephalometric radiograph after bimaxillary orthognathic surgery. (f) Final lateral cephalometric radiograph after secondary genioplasty and completion of treatment.

The chronological sequence of the interdisciplinary treatment is summarized in Table 3. Following the initial consultation and diagnostic evaluation, presurgical orthodontic treatment was initiated to achieve dental decompensation. Surgical planning was subsequently performed, followed by bimaxillary orthognathic surgery. Postoperative follow-up assessments confirmed satisfactory healing and treatment progression. Six months after surgery, osteosynthesis plates were removed, and a secondary genioplasty was performed to further optimize facial esthetics. An adjunctive 2-mm esthetic gingivectomy was subsequently carried out, increasing the clinical crown length of the maxillary central incisors from 7 mm to 9 mm, improving gingival contour and symmetry, with healthy periodontal tissues and stable soft-tissue architecture throughout follow-up. Orthodontic treatment was completed after 24 months, and retention was achieved using bonded lingual retainers and removable clear retainers. Long-term evaluation at three years and eight months demonstrated maintenance of the achieved skeletal, occlusal, esthetic, and periodontal outcomes, with no clinically evident relapse (Figures 17a-17f).

**Table 3:** Timeline of interdisciplinary treatment and follow-up.

Date	Clinical event
September 2020	Initial consultation and diagnosis: Adult female patient presenting with severe excessive gingival display
October 2020	Presurgical orthodontic treatment initiated: Dental decompensation
October 2021	Surgical planning presurgical: Study models and surgical splint preparation
November 2021	Bimaxillary orthognathic surgery: Le Fort I maxillary impaction and mandibular advancement
January 2022	Two-month follow-up
May 2022	Osteosynthesis plate removal and secondary genioplasty
September 2022	Esthetic gingivectomy
October 2022	Completion of orthodontic treatment; bonded lingual retainers placed and removable clear retainers delivered
July 2025	Final follow-up (three years and eight months)

## Discussion

The correction of severe EGD remains a clinical challenge because of its frequently multifactorial etiology. In the present case, the coexistence of vertical maxillary excess, a hyperdivergent skeletal Class II pattern, a short upper lip, and localized gingival hypertrophy justified an interdisciplinary treatment strategy combining orthodontics, orthognathic surgery, genioplasty, and periodontal management. Addressing each etiologic component was essential to achieve a harmonious and stable functional and esthetic outcome. These observations are consistent with previous reports emphasizing the importance of multidisciplinary management in patients with multifactorial EGD [5,6].

For patients with significant vertical maxillary excess, Le Fort I maxillary impaction remains the treatment of choice. In the present case, 5 mm of superior maxillary impaction effectively reduced EGD from 8 mm to ≤3 mm while promoting mandibular autorotation, contributing to correction of the skeletal Class II relationship and improvement of facial balance. This was reflected by objective cephalometric improvements, including a reduction in ANB from 9° to 5° and GoGn/SN from 48° to 43°. These findings are consistent with previous studies demonstrating the effectiveness and stability of orthognathic surgery in the management of EGD associated with dentofacial deformities [8]. Although surgery-first protocols have gained increasing popularity, the conventional sequential approach adopted in this case allowed rigorous dentoalveolar decompensation, optimized surgical planning, and progressive stabilization of the skeletal bases before esthetic refinements were undertaken [10].

Facial harmony was further enhanced by secondary advancement and downward genioplasty. Although mandibular advancement improved the sagittal skeletal relationship, residual chin retrusion persisted after bimaxillary surgery. Secondary genioplasty provided additional improvement in chin projection, lower facial balance, and cervicomental contour, complementing the skeletal correction achieved by orthognathic surgery. These findings are consistent with the role of adjunctive genioplasty in optimizing facial esthetics in selected patients with dentofacial deformities [8].

Management of the periodontal component represented another key aspect of treatment. Despite successful correction of the skeletal component by maxillary impaction, localized gingival hypertrophy persisted, partially covering the anatomical crowns and resulting in short clinical crowns. Consequently, a 2-mm gingivectomy was performed, increasing the clinical crown length of the maxillary central incisors from 7 mm to 9 mm while improving gingival contour, dentogingival harmony, and smile esthetics. Unlike lip repositioning procedures, which primarily target upper lip muscular hyperactivity [11], gingivectomy directly addresses the dentogingival component when periodontal conditions are favorable, allowing additional esthetic refinement without resorting to more invasive soft-tissue surgery [7].

The favorable stability observed at the three-year and eight-month follow-up highlights the importance of addressing all etiologic components of EGD, including skeletal, dental, soft-tissue, and periodontal factors. Stable skeletal, occlusal, periodontal, and esthetic outcomes were maintained throughout follow-up, which may be attributed to the documented long-term stability of maxillary impaction performed with rigid internal fixation, together with the strict retention protocol implemented after treatment [8].

Beyond the objective clinical, periodontal, and cephalometric improvements, treatment resulted in a marked enhancement of the patient's quality of life. The OQLQ score decreased from 86/88 before treatment to 4/88 after completion of treatment, with the greatest improvements observed in facial esthetics and social functioning. These findings highlight the substantial psychosocial benefits of comprehensive interdisciplinary management and are consistent with previous studies demonstrating significant improvements in quality of life following treatment of dentofacial deformities.

Nevertheless, the findings of this single-case report should be interpreted with caution, as they cannot be generalized to all patients presenting with multifactorial EGD. Although objective clinical, cephalometric, periodontal, and patient-reported outcome measures were included, further studies involving larger patient cohorts are required to confirm the long-term predictability and reproducibility of this interdisciplinary treatment approach.

## Conclusions

Within the limitations of a single-case report, the combination of presurgical orthodontics, bimaxillary orthognathic surgery, secondary genioplasty, and adjunctive gingivectomy appeared effective for the management of severe EGD of multifactorial etiology. This case highlights that successful treatment depends on accurate identification and individualized management of all contributing skeletal, soft-tissue, and periodontal etiologic factors rather than correction of a single component alone. The favorable stability observed at the three-year and eight-month follow-up further supports the value of a comprehensive interdisciplinary treatment approach. Nevertheless, further studies are required to confirm the long-term predictability of this treatment strategy.
